# Remote disruption of intestinal homeostasis by *Mycobacterium abscessus* is detrimental to *Drosophila* survival

**DOI:** 10.1038/s41598-024-80994-y

**Published:** 2024-12-28

**Authors:** Hamadoun Touré, Nicolas Durand, Vincent Rincheval, Fabienne Girard-Misguich, Isabelle Guénal, Jean-Louis Herrmann, Sébastien Szuplewski

**Affiliations:** 1 Infection et Inflammation, Université Paris-Saclay, UVSQ, INSERM, 78180 Montigny-Le-Bretonneux, France; 2 Université Paris-Saclay, UVSQ, LGBC, 78000 Versailles, France; 3 Assistance Publique-Hôpitaux de Paris, Hôpitaux Universitaires Ile-de-France Ouest, GHU Paris-Saclay, Hôpital Raymond Poincaré, 92380 Garches, France; 4 Present Address: Basic Sciences Division, Fred Hutchinson Cancer Center, Seattle, Washington USA

**Keywords:** Intestinal stem cells, Bacteriology

## Abstract

*Mycobacterium abscessus* (Mabs), an intracellular and opportunistic pathogen, is considered the most pathogenic fast-growing mycobacterium, and causes severe pulmonary infections in patients with cystic fibrosis. While bacterial factors contributing to its pathogenicity are well studied, the host factors and responses that worsen Mabs infection are not fully understood. Here, we report that Mabs systemic infection alters *Drosophila melanogaster* intestinal homeostasis. Mechanistically, Mabs remotely induces a self-damaging oxidative burst, leading to excessive differentiation of intestinal stem cells into enterocytes. We demonstrated that the subsequent increased intestinal renewal is mediated by both the Notch and JAK/STAT pathways and is deleterious to *Drosophila* survival. In conclusion, this work highlights that the ability of Mabs to induce an exacerbated and self-damaging response in the host contributes to its pathogenesis.

## Introduction

Mycobacteria are among the most primitive bacteria, and form a widely heterogeneous group of saprophytic, strictly pathogenic and opportunistic pathogen species^1,2^. While all known strict human and animal pathogens are slow-growing mycobacteria (e.g. *M. tuberculosis*), the group of fast-growing mycobacteria consists of saprophytic species with a small proportion of opportunistic pathogens such as *M.* *abscessus* (Mabs)^3^. The latter mainly causes mucocutaneous infections in humans, and severe pulmonary infections, particularly in patients with pre-existing lung structural and/or functional disorders, such as cystic fibrosis patients^4,5^. Compared to other fast-growing mycobacteria, Mabs possesses unique traits in terms of host colonization and mechanisms leading to its pathogenicity^6,7^. As a result, it is considered the most pathogenic fast-growing mycobacterium, but also the most worrisome due to its resistance to antibiotic and antituberculosis treatments^8^.

Mabs possesses the propensity to manipulate the host immune system using different strategies such as the inhibition of phagosome acidification in professional phagocytes^9^ and the formation of large mycobacterial aggregates (cords) that phagocytes are unable to internalize^10^. We have recently identified features of host colonization by Mabs, that are absent in other fast-growing species but shared with pathogenic slow-growing mycobacteria. Indeed, rapid internalization by, and growth in professional phagocytes protect Mabs from the antimicrobial peptide-mediated humoral response^11^. Moreover, Mabs is able to resist the lysis of infected phagocytes by immune cytotoxic cells^12^, such as NK cells, resulting in the depletion of the phagocyte cell pool capable of controlling the bacterium. This deleterious defense mechanism has been demonstrated in *Drosophila melanogaster* and confirmed in mice. As such, *Drosophila* is a well-established model for deciphering the spatiotemporal dynamics of the innate immune response against pathogens, including mycobacteria^13,14^.

Most studies have established the *Drosophila* intestine as a critical sentinel organ in the response to infections. Indeed, the *Drosophila* intestine is a sensor of bacterial products (*e.g.,* peptidoglycan and uracil) with an immediate impact on inter-organ communication^15,16^. The *Drosophila* intestine consists of a monolayer of epithelial cells resting on a thin layer of muscle cells and comprises five main cell types: the intestinal stem cells (ISCs), enteroendocrine cells (EEs), and their progenitors, the enteroblasts (EBs), and enterocytes (ECs)^17^. Intestinal homeostasis is mainly maintained by ISCs. Their physiological status is influenced by both intrinsic and extrinsic factors^18^. Once activated, they can differentiate into structural and specialized epithelial cells, most often after intestinal injury, to replace damaged or dead cells^19^. ECs, the differentiated structural cells, represent the major cell population in the epithelium. Their precursors are EBs, an intermediate stage between ISCs and ECs. EEs are also differentiated cells that, as their name suggests, can produce hormones or antimicrobial peptides depending on the context^20^.

As the gut is a sentinel organ in *Drosophila* for the perception of infection signals, we hypothesized that Mabs infection could alter gut homeostasis by inducing damages associated with an exacerbated epithelial response. Our study reveals that Mabs-infected flies exhibit a detrimental intestinal oxidative burst, and a deleterious higher rate of ISC differentiation into EC, through the activation of both JAK/STAT and Notch pathways. This work highlights the ability of Mabs to weaken its host by inducing self-damaging responses.

## Results

### Mabs systemic infection alters intestinal homeostasis by increasing ISC differentiation into EC

**Fig. 1:**
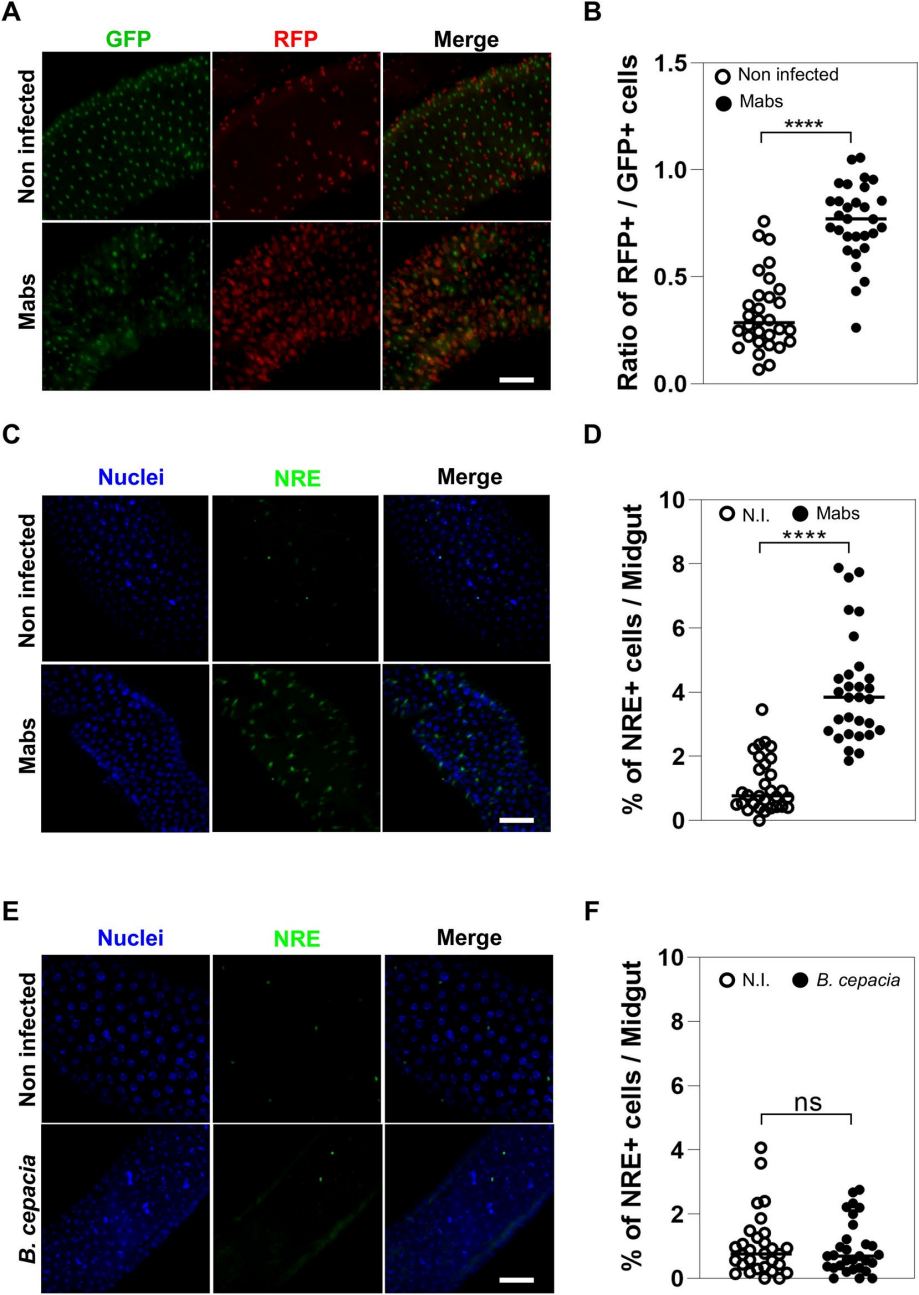
Mabs systemic infection disrupts *Drosophila* intestinal homeostasis. (**A**-**F**) (**A**) Representative confocal microscopy images of midgut of *esg* > *G-TRACE* flies on day 3 after injection with water (Non infected) or 500 CFU of Mabs (Mabs). (**B**) Comparison of ratios of RFP-positive to GFP-positive cells in *esg* > *G-TRACE* flies on day 3 p.i. after injection with water (Non infected) or 500 CFU of Mabs (Mabs). (**C**) Representative confocal microscopy images of midgut of *NRE-GFP* flies on day 3 p.i. injection with water (Non infected) or 500 CFU of Mabs (Mabs) (**D**) Comparison of the percentages of GFP-positive cells among the total number of cells in the midgut of *NRE-GFP* flies on day 3 p.i. after injection with water (Non infected) or 500 CFU Mabs (Mabs). (**E**) Representative confocal microscopy images of midgut of *NRE-GFP* flies on day 1 p.i. after water injection (Non infected) or 500 CFU of *B. cepacia*. (**F**) Comparison of the percentages of GFP-positive cells among the total number of cells in the midgut of *NRE-GFP* flies on day 1 p.i. after injection with water (Non-infected) or 500 CFU of *B. cepacia*. Statistical tests were performed using Student’s t-test (****p < 0.0001; ns: non-significant). Scale bars represent 10 µm.

To assess whether *M. abscessus* systemic infection could affect intestinal epithelial homeostasis, we used the Gal4-regulated lineage-tracing system (G-TRACE) system^21^ to follow the differentiation of ISCs. Indeed, the G-TRACE system allows the lineage-tracing of cells thanks to the production of fluorescent protein reporters for both real-time (RFP) and lineage-based (GFP) expression of the driver gene. The *escargot* (*esg*) gene is known to be expressed in *Drosophila* ISCs and EBs; we therefore used *esg-Gal4* as a driver line to express the *UAS-G-TRACE* transgene. We have observed a higher number and ratio of newly differentiated cells (RFP +) among the total cell number (GFP +) in the midgut of Mabs-infected flies compared to water-injected control flies (Fig. 1A-B). The morphology and nucleus size of the newly differentiated cells (RFP +) indicated that these cells were ECs (Fig. 1A), suggesting that their increased number could be due to the one of EBs. To test this hypothesis, we used a reporter fly line for the expression of *Notch-Responsive Element* (*NRE-GFP*), known to be expressed in EBs, the precursors of ECs^22^. We observed a significant increase in the number and ratio of NRE-positive cells in the midgut of Mabs-infected flies compared to water-injected control (Fig. 1C-D), supporting that Mabs infection increases ISC differentiation into ECs.

These results led us to wonder whether the increased differentiation of ISCs we observed was a common *Drosophila* response to any systemic infection or whether it was a feature of Mabs infection. We therefore counted the number of EBs in the midgut of NRE-GFP flies infected with the extracellular Gram-negative bacterium *Burkholderia cepacia*, which is known to be pathogenic to *Drosophila*^23^. Similar numbers of NRE-positive cells were counted in midgut whether flies were injected with water or *B. cepacia* (Fig. 1E-F), suggesting that unlike Mabs, systemic infection with *B. cepacia* does not induce differentiation of ISCs into ECs.

Collectively, these results suggest that Mabs systemic infection disrupts *Drosophila* intestinal homeostasis by triggering the excessive differentiation of ISCs into ECs.

### Notch-dependent ISC differentiation decreases the survival of Mabs-infected flies

**Fig. 2:**
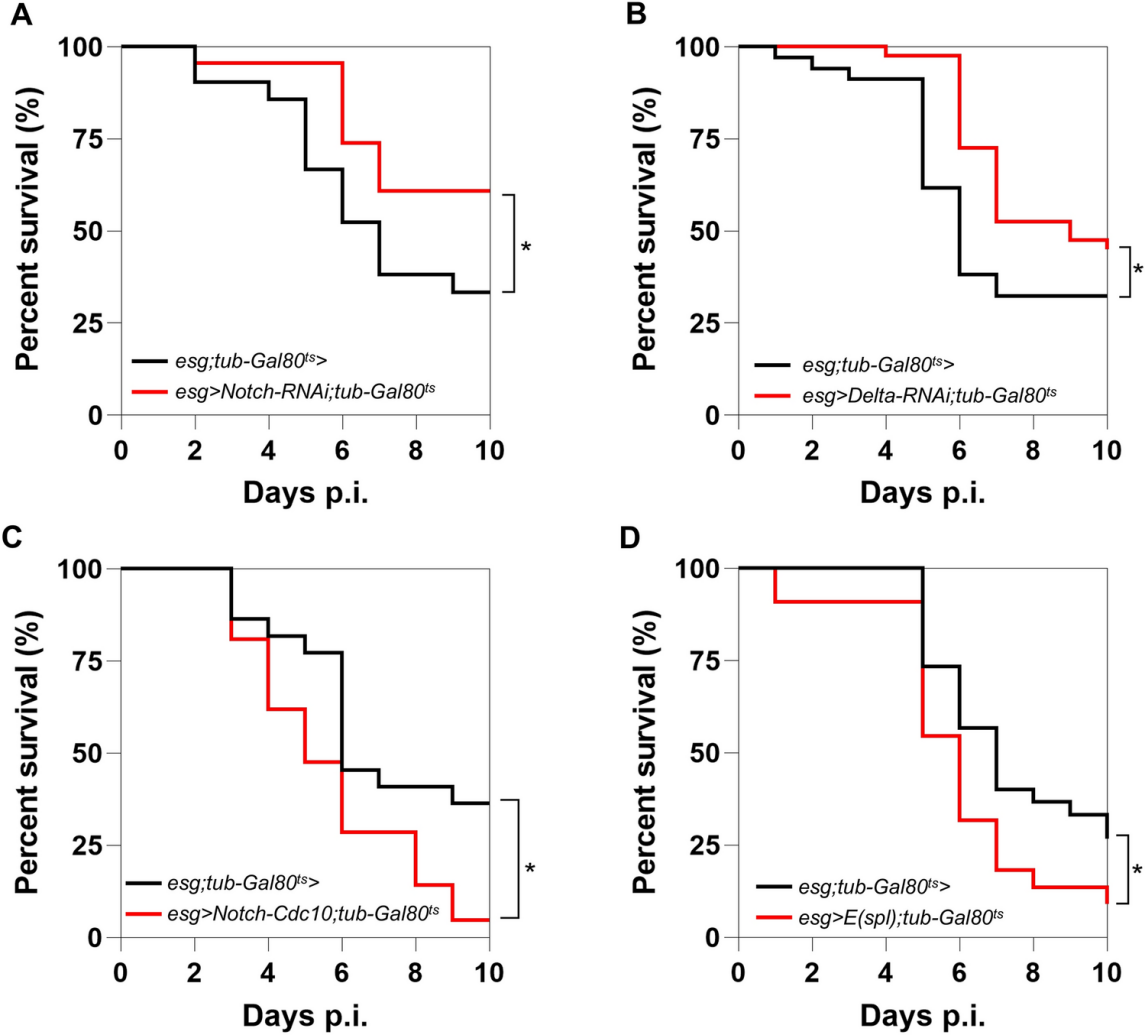
Notch-dependent ISC differentiation decreases *Drosophila* survival during Mabs infection. (**A-D**) Survival of *esg* > *;tub-Gal80*^*ts*^ and (**A**) *esg* > *Notch-RNAi;tub-Gal80*^*ts*^, (**B**) *esg* > *Delta-RNAi;tub-Gal80*^*ts*^, (**C**) *esg* > *Notch-Cdc10;tub-Gal80*^*ts*^, (**D**) *esg* > *E(spl);tub-Gal80*^*ts*^ following infection with 10 CFU of Mabs. Survivals were analyzed on 40–60 flies per condition using log-rank test (*p < 0.05).

Next, we assessed the influence of ISC differentiation induced by systemic infection with Mabs on the survival of infected flies. Therefore, we modulated the Notch signaling pathway, which has been implicated in ISC division and differentiation^24^. Since this signaling pathway is required for many developmental processes, we expressed transgenes using *esg-Gal4* as a driver only at the adult stage, thanks to the TARGET system^25^, to avoid any developmental bias. Surprisingly, depletion of *Notch* transcripts led to increased survival of flies following Mabs systemic infection compared to the control (Fig. 2A). We observed a similar increase in survival of infected flies by depleting transcripts encoding Delta, a Notch ligand (Fig. 2B). These results suggest that limiting Notch signaling, and thus limiting the activation of ISC differentiation into ECs, increases fly survival against Mabs systemic infection.

Therefore, we hypothesized that overactivation of the Notch pathway in the ISC should, conversely, decrease the survival of Mabs-infected flies. To test this hypothesis, we specifically expressed, in adult *Drosophila* progenitor cells (EBs + ISCs), transgenes encoding respectively an active intracellular truncated form of Notch (Notch-Cdc10)^24^ or the transcription factor Enhancer of split-m7 (E(spl)m7-HLH)^26^, a direct effector of the Notch pathway. As expected, the expression of either transgene decreased *Drosophila* survival after Mabs infection compared to the control (Figs. 2C-D).

Taken together, these results indicate that Notch-dependent differentiation of ISCs is deleterious to *Drosophila* survival against Mabs systemic infection.

We then sought to identify the mechanisms underlying this deleterious response.

### Mabs systemic infection induces deleterious ROS production by the enterocytes

Excessive proliferation of ISCs can be induced by oxidative response to infection^27,28^. Indeed, the detection of pathogenic bacteria-associated molecules, such as uracil and peptidoglycan, by ECs activates a reactive oxygen species (ROS) production mediated by Dual oxidase (Duox), an NAD(P)H oxidase^29^, that can eliminate the pathogens but also damage the intestinal epithelium. Therefore, we hypothesized that systemic infection with Mabs might induce increased ROS production in the gut, accounting for the observed deleterious excessive epithelial renewal.

**Fig. 3:**
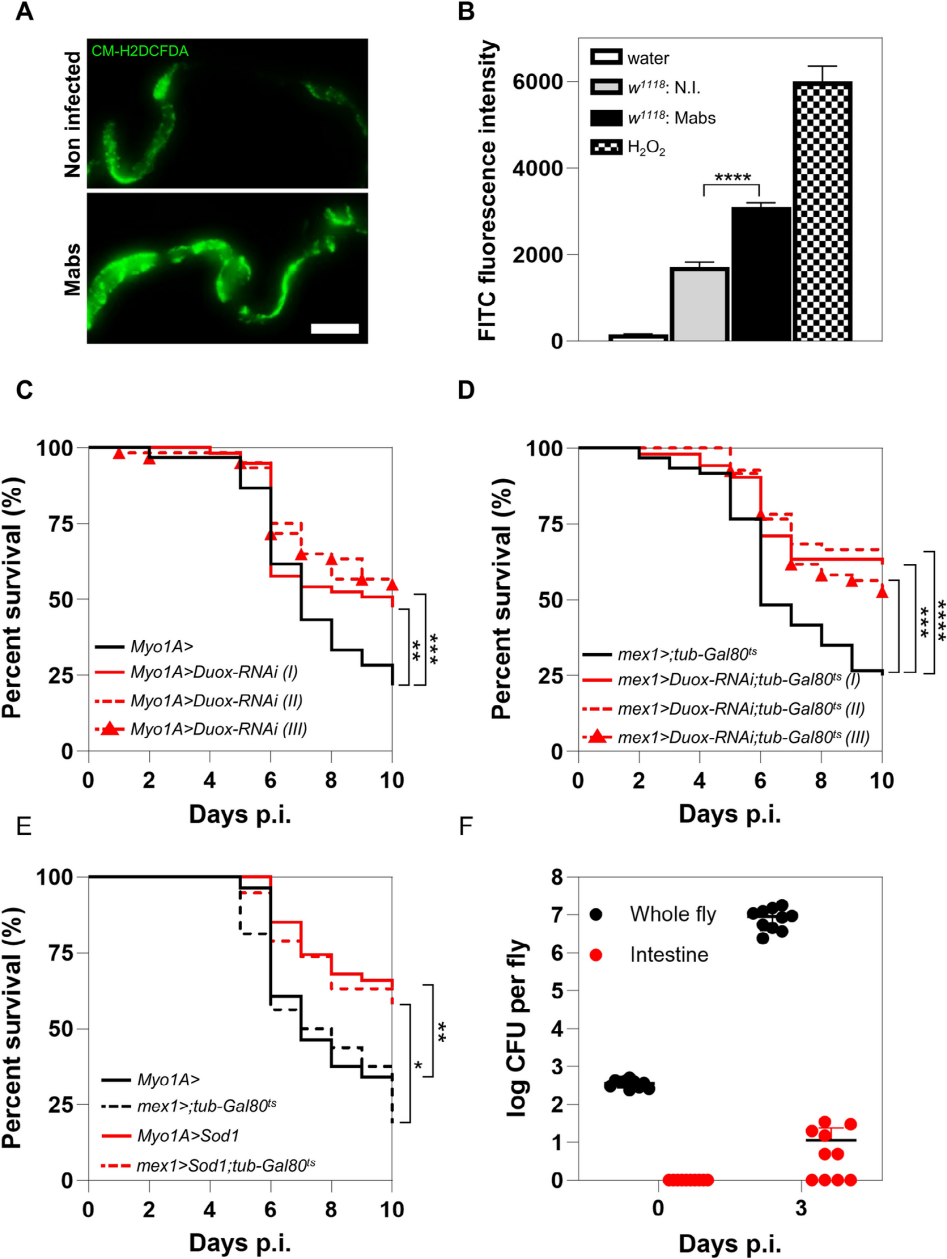
Mabs systemic infection induces deleterious ROS production by *Drosophila* enterocytes. (**A**-**F**) (**A**) Representative full-field confocal microscopy images of CM-H2DCFDA-labeled midgut of *w*^*1118*^ flies on day 3 p.i. after injection with water (Non infected) or 500 CFU of Mabs (Mabs). (**B**) Quantification of mean FITC fluorescence emitted by water (negative control), hydrogen peroxide (H_2_O_2_ – positive control) or 5 pooled intestines of *w*^*1118*^ flies injected with water (*w*^*1118*^:N.I.) or with 500 CFU of Mabs (*w*^*1118*^;Mabs) on day 3 p.i. after incubation with CM-H2DCFDA (**C**) Survival of *Myo1A* > and *Myo1A* > *Duox-RNAi* flies after infection with 10 CFU of Mabs. (**D**) Survival of *mex-1* > *;tub-Gal80*^*ts*^ and *mex-1* > *Duox-RNAi;tub-Gal80*^*ts*^ flies after infection with 10 CFU of Mabs. (**E**) Survival of *Myo1A* > *, mex-1* > *;tub-Gal80*^*ts*^, *Myo1A* > *Sod1* and *mex-1* > *Sod1;tub-Gal80*^*ts*^ flies after infection with 10 CFU of Mabs. (**F**) Mabs quantification in lysates of whole *w*^*1118*^ flies or in lysates of dissected intestines on day 0 and 3 days following infection with 500 CFU of Mabs. Survivals were analyzed on 40–60 flies per condition using log-rank test, and the mean fluorescence, by student *t-test*. (*p < 0.05; **p < 0.01; ***p < 0.001; ****p < 0.0001). Scale bar represents 500 µm. (I, II and III are three different RNAi lines targeting the *Duox* transcript).

We first assessed whether Mabs systemic infection could increase intestinal ROS production, using CM-H2DCFDA, a fluorescent reporter that labels ROS. As shown in the whole intestine (Fig. 3A) and quantified in intestinal lysates (Fig. 3B), Mabs-infected flies produced more intestinal ROS than the water-injected controls. This result suggested that systemic infection with Mabs triggers local ROS production in the intestine.

We next tested whether this increase in ROS production could be deleterious to infected flies by depleting the transcripts encoding the Duox enzyme specifically in ECs. This was done by using previously validated RNAi-encoding transgenes^30,31^. Increased survival was observed with the three RNAi lines driven by *Myo1A-Gal4*, an EC-specific driver, compared to control flies (Fig. 3C). Similar results were observed using another driver, *mex1-Gal4*, which allows expression specifically in ECs, and only at the adult stage thanks to the TARGET system (Fig. 3D). These results support that ROS produced by Duox in ECs are deleterious to the survival of Mabs-infected flies. As a control, similar survival was observed between control flies and *Duox* transcript-depleted flies during *B. cepacia* infection, suggesting that the protective survival conferred by *Duox* depletion is not common to any systemic infection in *Drosophila*, but is a feature of Mabs infection (Figure S1A).

We confirmed the deleterious role of the ROS production by ECs during Mabs infection by counteracting the oxidative response. Expression in ECs of a transgene encoding the antioxidant enzyme Superoxide dismutase 1 (Sod1), which is involved in the detoxification of superoxide ions and free radicals, increased the survival of Mabs-infected flies compared to the control (Fig. 3E). Similar results were obtained with two EC-specific drivers, one of which (i*.e., mex1-Gal4*) was specifically expressed in adults by the TARGET system (Fig. 3E).

Taken together, these results demonstrated that systemic infection with Mabs leads to a Duox -dependent intestinal oxidative burst that is detrimental to fly survival.

Since Duox-dependent ROS production by ECs is activated by the detection of pathogenic bacteria-associated molecules, such as uracil and peptidoglycan, in the intestinal lumen^28^, we wondered whether we could have injected Mabs directly into the intestine. We counted the number of bacteria in whole flies and dissected intestines immediately after the nano-injection of 500 colony-forming units (CFU) of Mabs (Fig. 3F). No mycobacteria were detected in the intestines, contrarily to whole flies, ruling out an intestinal Mabs injection or contamination. Since we observed ISC differentiation into ECs on day 3 p.i., we hypothesized that Mabs could damage the intestine by colonizing the epithelium. We thus counted mycobacterial CFU on day 3 p.i. either in whole flies or the intestine. Mabs actively multiplied in the whole fly, and few bacteria were present in the intestine (Fig. 3F), suggesting that Mabs could have infected the intestine.

### Remote activation of the JAK/STAT pathway by phagocytic plasmatocytes disrupts *Drosophila* intestinal homeostasis upon Mabs infection

Importantly, JAK/STAT-dependent differentiation of ISCs is a major consequence of the gut-damaging oxidative response upon oral infection with pathogenic bacteria^16,27^. During intestinal regeneration, the JAK/STAT pathway is activated to orchestrate the differentiation of ISCs into EBs^32,33^. This led us to hypothesize that the JAK/STAT pathway might be activated in the fly intestine during Mabs systemic infection, especially since we observed both epithelial regeneration and an exacerbated oxidative response in ECs.

**Fig. 4:**
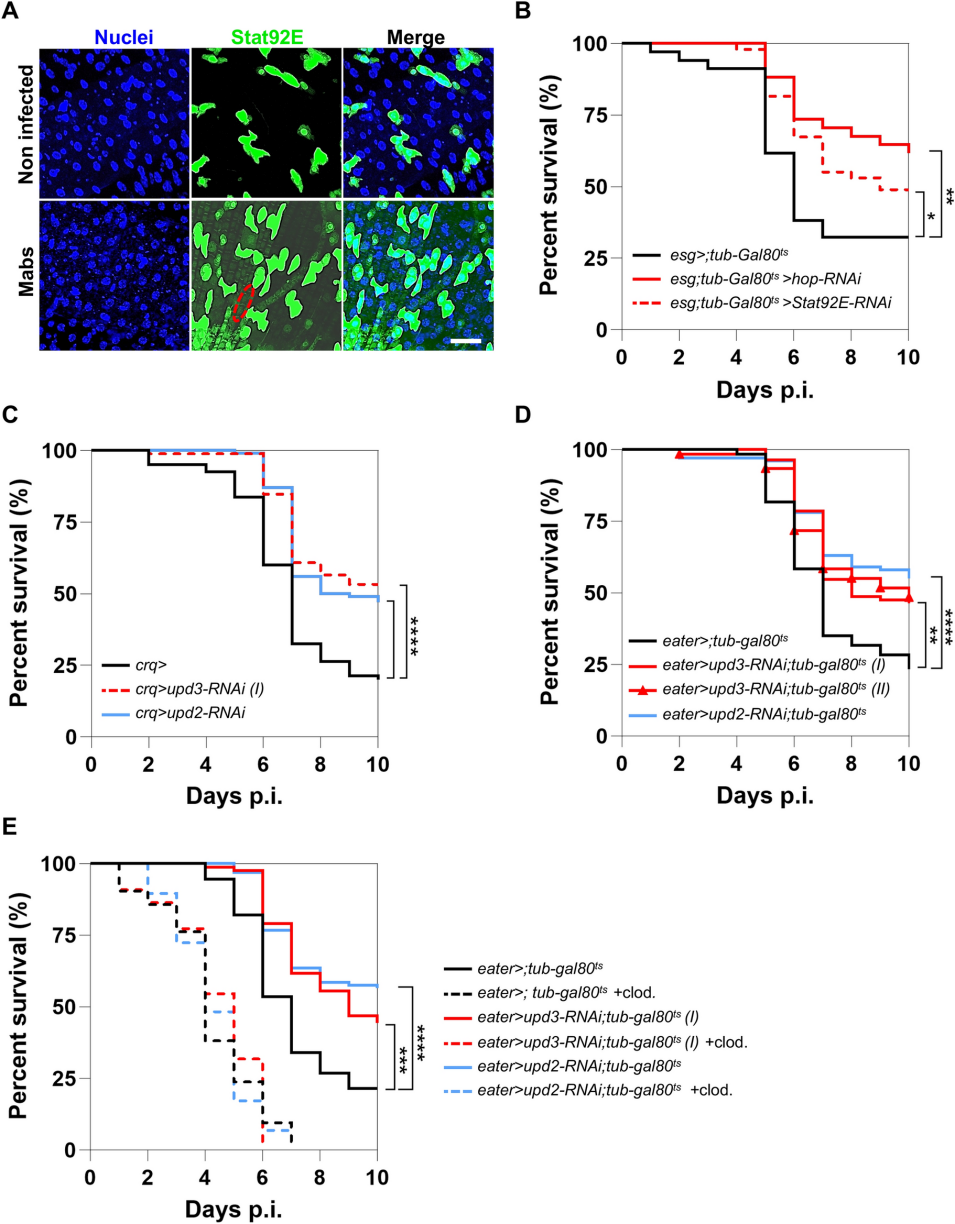
Remote activation of the JAK/STAT pathway by phagocytes disrupts *Drosophila* intestinal homeostasis upon Mabs infection. (**A**-**E**) (**A**) Representative confocal microscopy images of midgut of *10XSTAT-GFP* flies on day 3 p.i. after injection of water (Non infected) or 500 CFU of Mabs (Mabs). Ellipse indicates intestinal muscle striae. (**B**) Survival of *esg* > *;tub-Gal80*^*ts*^, *esg* > *hop-RNAi;tub-Gal80*^*ts*^, *esg* > *Stat92E-RNAi;tub-Gal80*^*ts*^ flies after infection with 10 CFU of Mabs. (**C**) Survival of *crq* > , *crq* > *upd3-RNAi* and *crq* > *upd2-RNAi* flies after infection with 10 CFU of Mabs. (**D**) Survival of *eater* > *;tub-Gal80*^*ts*^, *eater* > *upd3-RNAi;tub-Gal80*^*ts*^, and *eater* > *upd2-RNAi;tub-Gal80*^*ts*^ flies after infection with 10 CFU of Mabs. (**E**) Survival of *eater* > *;tub-Gal80*^*ts*^, *eater* > *upd3-RNAi;tub-Gal80*^*ts*^, and *eater* > *upd2-RNAi;tub-Gal80*^*ts*^ flies pre-injected with water or clodronate liposomes (+ clod.) 1 day before infection with 10 CFU of Mabs. Survivals were analyzed on 40–60 flies per condition using log-rank test (*p < 0.05; **p < 0.01; ****p < 0.0001). Scale bar represents 20 µm. (I and II are two different RNAi lines targeting the *upd3* transcript).

To test this hypothesis, we used flies carrying a transgenic fluorescent reporter of a STAT-dependent transcriptional activity (*10XSTAT-GFP*)^34^. GFP production was observed in the visceral muscle (dotted ellipse) of Mabs-infected flies, while no signal was detected in the muscle of uninfected control (Fig. 4A), suggesting that Mabs systemic infection activates the JAK/STAT pathway in the midgut, particularly in the progenitor cells and the visceral muscle.

This prompted us to test whether reducing JAK/STAT signaling in progenitor cells could increase *Drosophila* survival during Mabs infection. To this end, we depleted, specifically in adult progenitor cells, the transcripts encoding the unique *Drosophila* homologs of JAK (*hopscotch* (*hop*)) or STAT (*Stat92E*). Depletion of either transcript resulted in increased fly survival as compared to control flies (Fig. 4B), supporting that JAK/STAT activation in progenitor cells increases *Drosophila* susceptibility to Mabs infection.

Remote control of ISC differentiation has been described in *Drosophila* upon septic injury performed with *Micrococcus luteus* or *Enterococcus faecalis*, both Gram-positive bacteria, or the Gram-negative bacterium, *Erwinia carotovora carotovora 15*^35^. This process activates the JAK/STAT pathway in regenerating intestine, via the blood immune cells (hemocytes). Indeed, hemocytes of infected flies release Upd2 and Upd3, which are ligands of the JAK/STAT pathway, leading to activation of this signaling pathway in both intestinal progenitor cells (i.e., ISCs and EBs) and visceral muscles^35^. Interestingly, mycobacteria-infected hemocytes strongly produce Upd3^36^. Since Mabs remains intracellular during the first 3 days of infection^12^, we thus hypothesized that Mabs could remotely activate the JAK/STAT pathway in the intestine through the production of Upd2 and Upd3 by the infected phagocytes. We then depleted either *upd2* or *upd3* transcripts specifically in phagocytic plasmatocytes using either the *croquemort-Gal4* driver (Fig. 4C) or the adult stage-specific *eater-Gal4; tub-al80* driver (Fig. 4D). An increase in survival of flies depleted of these transcripts was observed, compared with the control (Fig. 4C-D). These results show that during Mabs infection, Upd2 and Upd3 production by phagocytic hemocytes is deleterious to fly survival, in contrast to *B. cepacia* infection (Figure S1B).

To confirm that infected phagocytes remotely activate the JAK/STAT pathway, we depleted chemically them using a protocol based on clodronate liposomes, that we had previously validated^12,37^. Our prediction was that Mabs infection in flies pre-injected with clodronate liposomes should not affect fly survival rate whether *upd2* or *upd3* transcripts are depleted or not. As expected, we observed that the depletion of phagocytes fully suppressed the increased survival conferred by *upd2* or *upd3* depletion (Fig. 4E), confirming that phagocytes are the main producers of Upd2 and Upd3 in Mabs-infected flies.

Altogether, these results support that phagocytic plasmatocytes control ISC differentiation remotely by producing Upd2 and Upd3 that activate the JAK/STAT pathway in ISCs and the visceral muscle.

## Discussion

In part due to its multiresistance to antibiotics, Mabs represents a major threat to cystic fibrosis patients, in whom it causes worrisome infections that are deleterious to respiratory function and its prevalence is constantly increasing^38–40^. Therefore, it becomes necessary to understand the aggravating host responses to Mabs infection to identify potential modular therapeutic targets that could alleviate the infection outcome. In this work, we demonstrated the ability of the Smooth (S) infective form of Mabs to induce a self-damaging response in host epithelium, here *Drosophila* intestine, through an oxidative burst leading to a deleterious excessive tissue renewal.

The ability to cause damage in the infected host, through the induction of exacerbated responses, is a well-known feature of the other morphotype of Mabs, the more inflammatory and virulent rough (R) form. R-Mabs results from an irreversible transition of S-Mabs during the infection, due to the loss of the surface glycopeptidolipids (GPL) in the latter. R-Mabs hypervirulence is in part related to the increased exposure of surface molecules in the absence of GPL, which then stimulates more strongly the host immune response^41^. Thus, exposure of surface lipoproteins results in an exacerbated TLR2 response and increased responses mediated by tumor necrosis factor (TNF) and interleukin-1β (IL-1β)^42–44^. This exacerbated inflammatory response is deleterious to respiratory function, and the presence of the R form is associated with higher respiratory failure and mortality in infected patients^45^.

Our work highlighted that S-Mabs systemic infection leads to ROS production in the *Drosophila* intestine and that this excessive oxidative response is detrimental to fly survival. So far, ROS production has mainly been assessed in vertebrate immune cells upon Mabs infection. Indeed, monocytes and macrophages are the most common human lung cells infected by Mabs^46^ and immune cells are amenable to ex vivo studies. Thus, Mabs infection triggers ROS production, and this oxidative environment promotes Mabs growth in mouse primary macrophages and THP1 cell line^47,48^. Moreover, DUOX2/NADPH-dependent ROS production by human neutrophils does not affect Mabs intracellular load^49^.

At the epithelial level, ROS act as a chemoattractant for the recruitment of immune cells at the infection site, as described in the zebrafish model^50^. Our work highlights another facet of ROS contribution in Mabs infection pathophysiology, namely a detrimental role when ROS production is exacerbated. Our observation that the excessive intestinal oxidative response is detrimental to fly survival is in line with the self-destructive production of disproportionately high amounts of ROS by (1) Mabs-infected human pulmonary epithelial organoids^51^, and (2) the lungs of tuberculosis patients^52^. As described in orally infected *Drosophila*, Duox-mediated ROS production, aimed at killing bacteria, can also damage and then lead to the death of ECs, the majority cells of the intestine and guarantors of the integrity of the epithelium^53,54^. Furthermore, ROS increase the permeability of the peritrophic matrix, the first line of protection in the lumen, at the expense of fly survival^16,28^. Tissue homeostasis is then maintained through the proliferation of ISCs and the differentiation of their daughter cells into new ECs by initiating the activation of the JAK/STAT pathway in progenitor cells and the visceral muscle, as we observed^33^.

In addition to ROS production, we observed that Mabs infection activates the JAK/STAT pathway in the gut. Our data seem to indicate that Mabs remotely induces Notch and JAK/STAT-mediated intestinal regeneration. In an infection context, the JAK/STAT pathway is activated after Upd2 and/or Upd3 binding to the transmembrane receptor Dome^55^. Upd3 can either be produced by hemocytes following septic injury or EC during oral infection^35,56^. By combining the facts that (1) we performed systemic infections, (2) Mabs grows in phagocytic hemocytes during the first three days of the infection^12^, (3) mycobacterial-infected phagocytes strongly produce Upd3^36^, and (4) we did not observe JAK/STAT activation following *B. cepacia* infection, the ISC differentiation is most likely remotely governed by Upd2 and Upd3 production by Mabs in infected phagocytes.

This model is further supported by the fact that hemocyte-derived Upd3 activates the JAK/STAT-dependent ISC proliferation only during systemic infection and not during oral infection^35^. Although, in this report JAK/STAT activation in progenitor cells is protective against the infection rather in our model it is deleterious^35^. This difference could be due to either the mode of infection (septic wound vs. nano-injection) or the type of pathogen (*Erwinia caratovora caratovora 15* (*Ecc15*/*Pectobacterium carotovorum*) vs. Mabs). Notably, *Ecc15* is a Gram-negative bacterium as well as *B. cepacia*, which we showed here to be unable to induce in the midgut neither ROS production nor ISCs differentiation. Further work is needed to explain this difference, as well as to identify Mabs virulence factors that stimulate Upd2 and Upd3 production by infected phagocytes.

## Materials and methods

### Bacterial strains and cultures

*M. abscessus subsp abscessus* smooth morphotype *(M.* *abscessus herein; ATCC 19,977)* and *Burkholderia cepacia* (clinical strain) were used in this study. Mabs was grown at 37 °C in Middlebrook 7H9 medium (Sigma-Aldrich, Saint-Louis, USA) supplemented with 1% glucose and glycerol 0.2% at aerobic condition until an OD_600_ between 0.6 and 0.8. *B.* *cepacia* was cultured in standard Luria–Bertani (LB) medium. Bacterial cultures were then centrifuged to get concentrated aliquots that were then frozen at -80 °C in 10% glycerol.

## *Drosophila* maintenance, crosses, and infection

Flies were raised on standard corn-agar medium at 25 °C. Crosses were performed at 25 °C. Transgene expression was performed using the *UAS-GAL4* system^57^. *Drosophila* stocks obtained from Dr M. Crozatier, Dr A. Bardin, Dr C. Mallart, Dr J. Montagne, and the Bloomington Drosophila Stock Center (NIH P40OD018537) were used in this study and are listed in Table 1.

**Table 1:** List of *Drosophila* strains used in this study.

Genotype	Source	Identifier
y[1] w[*];P{w[+mC]=crq-GAL4}2	Bloomington Drosophila Stock Center (BDSC)	25041
*w[1118]; P{w[+mC]=eater-GAL4.K}3-1*	BDSC	36321
*y[1] v[1]; P{y[+t7.7] v[+t1.8]=TRiP.JF02867}attP2*	BDSC	28032
y[1] w[*]; P{w[+mC]=UAS-E(spl)m7-HLH.C}2	BDSC	26681
w[1118]; P{w[+m*]=NRE-EGFP.S}5A	BDSC	30727
w[*]; sna[Sco]/CyO; P{w[+mC]=tubP-GAL80[ts]}ncd[GAL80ts-7]	BDSC	7018
w[*]; P{w[+mC]=UAS-RedStinger}4, P{w[+mC]=UAS-FLP.D}JD1, P{w[+mC]=Ubi-p63E(FRT.STOP)Stinger}9F6/CyO	BDSC	28280
*y[1] v[1]; P{y[+t7.7] v[+t1.8]=TRiP.HM05061}attP2 *		
(*upd3-RNAi* I)	BDSC	28575
y[1] sc[*] v[1] sev[21]; P{y[+t7.7] v[+t1.8]=TRiP.HMS00646}attP2		
(*upd3-RNAi *II)	BDSC	32859
y[1] sc[*] v[1] sev[21]; P{y[+t7.7] v[+t1.8]=TRiP.HMS00901}attP2		
(*upd2-RNAi*)	BDSC	33949
*y[1] v[1]; P{y[+t7.7] v[+t1.8]=TRiP.GL00678}attP40*		
(*Duox-RNAi* I)	BDSC	38907
y[1] sc[*] v[1] sev[21]; P{y[+t7.7] v[+t1.8]=TRiP.HMS00692}attP2		
(*Duox-RNAi* II)	BDSC	32903
y[1] sc[*] v[1] sev[21]; P{y[+t7.7] v[+t1.8]=TRiP.GL00688}attP40		
(*Duox-RNAi* III)	BDSC	38916
*w*^*1118*^	Dr F. Rouyer (from NIG fly)	N/A
*mex1-Gal4;tub-Gal80ts*	Dr Jacques Montagne	N/A
*Myo1A-Gal4*	Dr Allison Bardin	NP0001
*yw;esg-Gal4/CyO;P{w[+mC]=tubP-GAL80[ts]* *P{UAS-GFP})*	Dr Allison Bardin	N/A
*UAS-Ncdc10(w;Kr[If]/CyO P[wg-lacZ];P[mw,UAS-Ncdc10]/TM6b Tb)*	Dr Allison Bardin	N/A
P{w[+mC]=UAS-N.dsRNA.P}14E,w[*]	Dr Allison Bardin	N/A
w[*]; TI{w[+mW.hs]=TI}mir-bft[Delta263a]/CyO, P{w[+mC]=GAL4-twi.G}2.2, P{UAS-2xEGFP}AH2.2	BDSC	58902
*10XSTAT92E-GFP*	Dr Charlotte Mallart	[34]
*UAS-hop-RNAi*	Dr Charlotte Mallart	VDRC 40037
*UAS-Stat92E-RNAi*	Dr Charlotte Mallart	VDRC 43866

Frozen bacterial aliquots were thawed on ice, homogenized with a 30-gauge insulin needle (Becton–Dickinson, France) to avoid clumps. Serial tenfold-dilutions were done and 30 μL of each dilution were spread on blood agar plate for mycobacteria (COS, bioMérieux, France) or on classic LB agar plate for *B.* *cepacia*. Plates were then stored at 37 °C for 2 or 3–7 days depending on bacteria and colony forming units (CFU) counts were determined.

Bacterial inoculum was diluted in water to get suitable concentrations. 5–7 days old virgin female flies were anesthetized with CO_2_ (Inject-Matic, Switzerland), and were infected with 50 nL of the suspension containing 10 bacteria by injection into the sternopleural suture. Infections were performed using a nano-injector Nanoject III (Drummond Scientific company, USA) charged with a calibrated pulled glass needle made with a DMZ-universal-electrode-puller (Zeitz instruments, Germany).

To chemically deplete the phagocytes, 24 h prior to infection with *M. abscessus*, flies were individually pre-injected with 69 nL of clodronate liposomes (Clodrosome CLD-8901, Encapsula, USA) diluted in PBS (ratio 1:5). Flies were kept anesthetized no more than 10 min. Infected flies were maintained at 28 °C in controlled humidity condition. Twenty flies were used for every experimental condition and each experiment was performed at least in two independent replicates. Mortality was registered daily, and surviving flies were transferred into a new vial every two days, until day 10 post-infection (p.i.).

### Bacterial load quantification

Ten individually dissected intestines or whole flies per experimental condition were individually grounded in 250 µL of water using sterile polypropylene cones (Kimble 749,521–1590, Kimble Chase, USA). The broths were centrifuged at 1,200 g for 2 min (min.) and diluted by tenfold serial dilutions. 50 μL of each dilution were spread on VCA3 plates (VCA3, bioMérieux, France) containing selective antibiotics for *M.* *abscessus* (Vancomycin, Colistin, Trimethoprim and Amphotericin B). The plates were kept at 37 °C for one week.

### Intestine dissection and microscopy

Thirty infected or uninfected *NRE-GFP*, *10XSTAT-GFP*, *esg* > or *esg* > *G-TRACE* flies were dissected in 1X PBS; intestines were then fixed in 3.7% PFA for 90 min at room temperature with agitation. Intestines were then rinsed with PBT (PBS 1X containing 0.1% Triton) twice for 10 min. and then stained with Hoechst diluted 1:500 in PBT (Invitrogen Hoechst 33,342). Stained intestines were equilibrated in PBS/glycerol (v/v) for at least 30 min. with shaking, then kept in the same solution overnight before being mounted between slide and coverslip in 15 µL of Citifluor (Electron Microscopy Sciences).

Images were acquired with a confocal microscope (Leica SP8 X Laser scanning confocal microscope), then processed with ImageJ (Bitplane). For *esg* > *G-TRACE* flies, ImageJ’s ITCN plugin was used to quantify RFP and GFP positive cells, with a threshold of 0.

### Quantification of intestinal ROS

Five flies from each experimental condition were dissected in 1X PBS, then the entire intestines were recovered, and the Malpighian tubes were cut and discarded as they are known to impact intestinal ROS quantities. The intestines were then grounded in 10 µM CM-H2DCFDA (Fisher Scientific, C6827), a FITC-labeled ROS reporter. Grinds were incubated for 30 min. at room temperature with agitation, then their fluorescence levels were read with a spectrophotometer (Tecan Infinite M200, Life Sciences) at FITC wavelengths (excitation at 495 nm, emission at 521 nm).

For microscopic visualization, the dissected intestines were incubated for 15 min. in 10 µM CM-H2DCFDA, mounted in Citifluor between slide and coverslip (Electron Microscopy Sciences) and then observed using a full-field automated microscope (IX83 microscope, Olympus) with the 10X objective. Images were reconstituted using the IMARIS software (Bitplane).

### Statistical analysis

All statistical tests were performed using GraphPad Prism 9.0.0 (GraphPad Software Inc., USA). The log-rank (Mantel-Cox) test for Kaplan–Meier survival curves was used to evaluate survival statistics significance. Mean fluorescence quantifications and RFP + /GFP + cells ratios between the different experimental conditions were compared by student *t-test*. *p* values inferior to 0.05 were considered significant.

## Supplementary Information

Supplementary Information.

## Data Availability

All data generated or analysed during this study are included in this published article (and its Supplementary Information files).
