# Supplementary material for: Remote disruption of intestinal homeostasis by Mycobacterium abscessus is detrimental to Drosophila survival
Source: Sci Rep. 2024 Dec 28;14:30775. doi: 10.1038/s41598-024-80994-y (PMC11681065; doi:10.1038/s41598-024-80994-y)
Supplement: Supplementary file 1 — Supplementary Information. [file 41598_2024_80994_MOESM1_ESM.pdf]

Supplemental Material Touré *et al.*

Figure S1

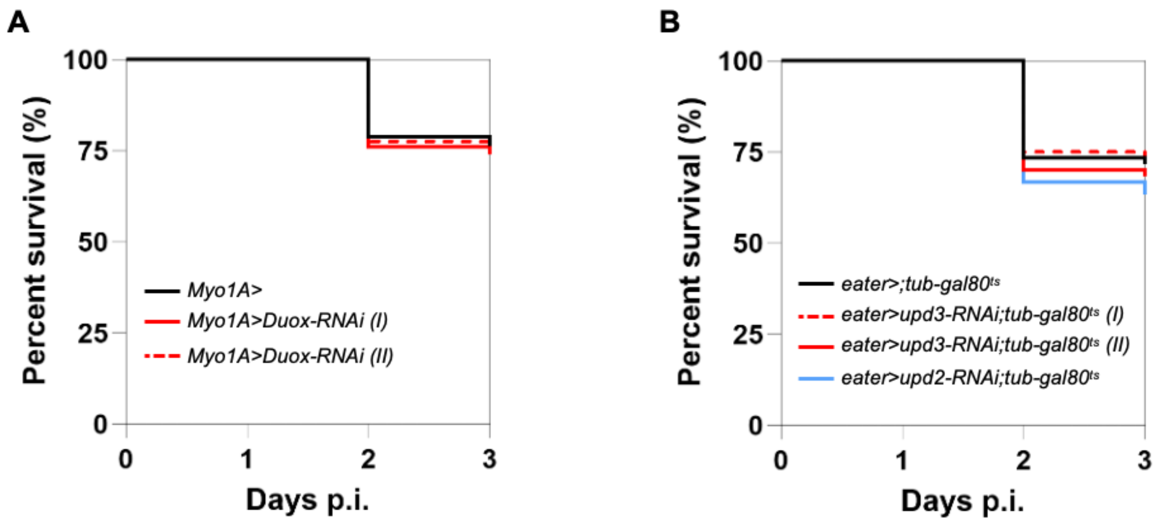

Figure S1. Interfering with intestinal ROS production or JAK/STAT signaling does not alleviate *Drosophila* mortality during *B. cepacia* infection.

(A-B) (A) Survival of *Myo1A>*, and *Myo1A>Duox-RNAi* flies after infection with 10 CFU of *B. cepacia*. **(B)** Survival of *eater>;tub-Gal80ts*, *eater>upd3-RNAi;tub-Gal80ts*, and *eater>upd2-RNAi;tub-Gal80ts* flies after infection with 10 CFU of *B. cepacia*. Survivals were analyzed on 40-60 flies per condition using log-rank test. (I and II are two different RNAi lines targeting the *Duox* transcript in A or *upd3* transcript in B).
